# Nociplastic Pain in Endometriosis: A Scoping Review

**DOI:** 10.3390/jcm13247521

**Published:** 2024-12-10

**Authors:** Avonae Gentles, Emma Goodwin, Yomna Bedaiwy, Nisha Marshall, Paul J. Yong

**Affiliations:** 1 Department of Obstetrics and Gynecology, University of British Columbia, Vancouver, BC V6H 3N1, Canada; avonae.gentles@cw.bc.ca (A.G.); nisha.marshall@cw.bc.ca (N.M.); 2 BC Women’s Center for Pelvic Pain and Endometriosis, Vancouver, BC V6H 3N1, Canada; emma.goodwin@cw.bc.ca (E.G.); yomna04@student.ubc.ca (Y.B.); 3 Women’s Health Research Institute, Vancouver, BC V6H 3N1, Canada

**Keywords:** endometriosis, nociplastic pain, central sensitization, peripheral sensitization, pain phenotyping

## Abstract

Endometriosis is an inflammatory chronic condition associated with nociceptive, neuropathic, and nociplastic pain. Central sensitization (CS) is the primary nociplastic pain mechanism. However, there are currently no standardized methods for detecting CS or nociplastic pain. This review aims to identify available tools for characterizing CS/nociplastic pain in endometriosis-related chronic pelvic pain. Following the PRISMA-P protocol, MEDLINE, Embase, Scopus, and PsychINFO databases were searched on 23 April 2024, for the terms “endometriosis”, “central sensitization”, “nociplastic pain”, “widespread pain”, and “assessment tools”. Publications were selected if they mentioned tool(s) for detecting nociplastic pain or CS in endometriosis patients. Information was extracted on study demographics, assessment types, and the tools used for detection. Of the 379 citations retrieved, 30 papers met the inclusion criteria. When working to identify CS and nociplastic pain, fourteen studies exclusively used patient-reported questionnaires, six used quantitative sensory testing (QST), two used clinical assessments, and eight used multiple approaches combining patient-reported questionnaires and clinical assessment. This review illustrates the diversity of tools currently used to identify CS and nociplastic pain in endometriosis patients. Further research is needed to evaluate their validity and to standardize methods in order to improve the accuracy of nociplastic pain identification and guide treatment.

## 1. Introduction

The goal of this scoping review is to assess the state of the literature regarding nociplastic pain in endometriosis. In 2021, the International Association for the Study of Pain (IASP) updated their definitions and clinical criteria for musculoskeletal pain [1]. One of the aims of this update was to provide more precise definitions of 3 pain types: nociceptive, neuropathic, and nociplastic pain [2]. According to the IASP, nociceptive pain refers to “pain that arises from actual or threatened damage to non-neural tissue and is due to the activation of nociceptors” [1]. This differs from neuropathic pain, which is described as “pain caused by a lesion or disease of the somatosensory nervous system” [1,2]. Nociplastic pain is a descriptive term used to characterize the pain phenotype that could not be exclusively defined as nociceptive or neuropathic [3]. In the 2021 definition, the IASP defines nociplastic pain as “[p]ain that arises from altered nociception despite no clear evidence of actual or threatened tissue damage causing the activation of peripheral nociceptors or evidence for disease or lesion of the somatosensory system causing the pain” [1,2]. Clinically, nociplastic pain is defined by four patient-reported requirements:

(1) A pain duration of at least 3 months;
(2) A regional pain distribution (i.e., not discrete);
(3) Pain that is not entirely explained by nociceptive or neuropathic mechanisms;
(4) Signs of pain hypersensitivity [2].

If a patient meets all four of these criteria, they are considered to have “possible nociplastic pain” [1,2]. However, if a patient meets these criteria, has a history of hypersensitivity in the region of pain, and one or more of the following comorbidities—increased sensitivity to sound, light, and/or odors, sleep disturbance, fatigue, or cognitive problems—they are considered to have “probable nociplastic pain” [2].

Notably, there is a lack of clinical criteria for definite nociplastic pain. This uncertainty in the definition of nociplastic pain has resulted in variability in its clinical interpretation and identification. Patients with conditions such as fibromyalgia, irritable bowel syndrome, and bladder pain syndrome are often characterized as having nociplastic pain. This can be attributed to heightened pain perception, which results in symptoms of chronic abdominopelvic pain and widespread musculoskeletal pain [3,4,5]. Chronic pain conditions tend to be highly heterogenous, with some patients experiencing more than one of these three types of pain [6]. To add further complexity, nociceptive inputs from peripheral tissues and neuropathic pain can also influence the development and persistence of central nervous system (CNS) sensitization [7,8].

Central nervous system sensitization (central sensitization or CS) describes the “amplification of neural signaling within the CNS that elicits pain hypersensitivity” and is the primary underlying mechanism in nociplastic pain [3,9]. CS can develop from different underlying causes. In some cases, peripheral damage can lead to the sensitization of the CNS in a way that persists over time but can be resolved with interventions that treat the peripheral cause. In other cases, CS is driven by a central component that is independent of peripheral damage, and thus does not respond to treatments that target this damage [7]. The peripheral sensitization of local nociceptors, which describes increased responsiveness at the primary site of pain (called primary hyperalgesia), can also occur, and this may lead to the development of, or exist alongside, CS [10,11]. However, while peripheral sensitization is a local pathology, one indicator of CS is an increased sensitivity to stimuli outside of the area where the primary injury or damage occurred (secondary hyperalgesia) [7,11]. Other clinical manifestations of CS include regional allodynia, defined as pain due to a normally non-painful stimulus [1,12]. CS has also been linked to psychological factors such as anxiety, panic attacks, and depression [13]. While some conditions, such as fibromyalgia, have been studied in relation to CS and treatment outcomes, others are still lacking research [7].

Endometriosis is an inflammatory chronic pain condition characterized by the growth of endometrial-like lesions outside of the uterus [14]. These lesions are predominantly found in the abdominopelvic organs and peritoneal cavity [15,16]. These endometrial-like tissues are hormonally regulated (estrogen-dependent), with a component of local inflammation contributing to nociceptive pain [15]. Increasing evidence also supports the role of multifactorial contributors to pain such as abdominal wall pain, pelvic floor myalgia, and higher scores on the pain catastrophizing scale in individuals with endometriosis [17]. Endometriosis-related pain can take several forms, including dysmenorrhea (painful menstruation), dyschezia (painful bowel movements), dysuria (painful urination), dyspareunia (painful sexual activity), and cyclical and noncyclic pelvic pain [18]. Endometriosis is known to be heterogenous, and patients often report a variety of outcomes for the same treatment [19]. Nociplastic pain may provide an explanation for some of this heterogeneity. However, there are currently no known standardized methods for characterizing nociplastic pain in endometriosis-specific populations. This gap presents a limitation in treating endometriosis-related chronic pelvic pain. As such, this review intends to elucidate the current tools that are being used to identify nociplastic pain in endometriosis pain populations and assess the quality of evidence in support of their use.

Historically, endometriosis-related pain was considered a result of local inflammation and irritation from lesions or nodules and was thus conceptualized as predominantly nociceptive [20]. Recent studies suggest nociceptive, neuropathic, and nociplastic mechanisms could all be associated with endometriosis-related pain [18]. Endometriosis-related neuropathic pain results from damage to the nerves surrounding endometriosis lesions or nodules [19]. CS and nociplastic pain are clinically relevant, as studies show that endometriosis patients with more symptoms of CS are more likely to experience worse pelvic pain symptoms, independent of endometriosis type or previous surgery [21]. In fact, endometriosis patients with clinical indicators or comorbidities commonly associated with CS have a worse quality of life and higher pain scores for chronic pelvic pain, deep dyspareunia, dyschezia, and back pain following surgical treatment for endometriosis [10,22,23]. Thus, the ability to clinically assess for nociplastic pain characteristics and the presence of CS is imperative. Moreover, the improved classification of nociplastic pain and CS in clinical settings could enable the implementation of more personalized pain treatments, targeting specific pain phenotypes and their multifactorial influences in people with endometriosis-related pain [7].

We conducted a scoping review to determine the scope of available tools and resources for characterizing CS in endometriosis patients and assess their quality. 

## 2. Scoping Review: Identifying Nociplastic Pain in Endometriosis

### 2.1. Rationale

Identifying patients with endometriosis who present with symptoms of CS, and consequently, nociplastic pain, is paramount to improving their quality of life and pain outcomes. While there are various questionnaires and assessments clinically used to detect nociceptive and neuropathic pain in endometriosis populations, there are currently no standardized methods of detecting nociplastic pain. This discrepancy may stem from the relative novelty of the nociplastic pain phenotype in the research community [9]. Nociplastic pain and CS exemplify neuroplasticity, occurring at cellular and functional levels, and often present unclear clinical features [2,8,24]. Combined with the subjective nature of pain and the frequently heterogeneous clinical presentations that overlap with psychological factors such as anxiety and depression, capturing instances of CS in a clinical setting is challenging [25,26]. Fortunately, there are an increasing number of methods for detecting and quantifying the degree of CS that may act as a proxy for nociplastic pain in chronic pain populations. For example, the Central Sensitization Inventory (CSI) has been validated and used to assess CS in endometriosis populations [27]. It provides an estimate of the impact of CS symptoms on a scale from 0 to 100. The CSI is able to distinguish between patients with chronic overlapping pain conditions (COPCs) and those with chronic pain conditions without a central pain component, as well as between patients with COPCs and healthy controls [28,29]. Additionally, in an endometriosis population, a 40-point cutoff has shown clinical significance [27], which the CSI correlating with worse pain outcomes following endometriosis surgery [23]. However, the CSI is only a proxy and may primarily be associated with psychological factors such as stress, depression, and anxiety [26]. Nonetheless, having a standardized and widely accepted clinical tool that can efficiently and effectively identify these patients is essential for treating endometriosis and managing pain.

Quantitative sensory testing (QST) is a semi-objective test that is frequently used to detect modulations in somatosensory processing in people with CS through a battery of sensory and pain threshold tests [30]. Another method includes using functional magnetic resonance imaging (fMRI) to detect changes in gray matter in areas of the brain related to pain sensation [31]. However, both these methods are resource-extensive and not feasible for use in clinical settings. Furthermore, ongoing research is needed to further validate these methods as an indicator of CS in endometriosis populations. As a result, there may be a subset of people living with endometriosis who also experience CS and nociplastic pain that remains unrecognized and untreated. In fact, within 5 years of standard endometriosis treatment, over 40% of patients will experience persistent or recurrent pain [32]. This scoping review investigates the range of questionnaires, tools, and clinical assessments being used to detect CS or nociplastic pain in populations with endometriosis-related pain. This review will provide an overview of current tools and methods of assessment being used to detect CS and nociplastic pain in an endometriosis pain population, along with supporting evidence. The findings from this review will inform future research seeking to determines the optimal clinical approach for identifying CS and nociplastic pain in endometriosis.

### 2.2. Methods

This scoping review adhered to the Preferred Reporting Items for Systematic Reviews and Meta-analysis extension for Scoping Review (PRISMA-ScR) (Table S2 in Supplementary Materials) [33]. Additionally, the protocol was created using the Preferred Reporting Items for Systematic Reviews and Meta-analysis Protocols (PRISMA-P) and was revised by the research team. The final protocol was registered on the Open Science Framework (OSF) (https://www.cos.io/ accessed on 12 April 2024) on 12 April 2024 and can be found at (https://doi.org/10.17605/OSF.IO/S358T) [34,35].

With the aid of a health science librarian, we conducted a systematic search of MEDLINE, Embase, Scopus, and PsychINFO on 23 April 2024. We used the following key terms: “endometriosis”, “central sensitization”, “nociplastic pain”, “widespread pain”, and “assessment tools”. The final search strategy used for MEDLINE can be found in Table S1 in the Supplementary Materials. Given the novelty of nociplastic pain in IASP’s characterization of pain, we did not apply any time limits. The final search was uploaded to Veritas Health Innovation (Melbourne, Australia) Covidence systematic review software (www.covidence.org accessed on 23 April 2024), an online platform that facilitates the production of systematic and other literature reviews [36]. We excluded published abstracts, editorial letters, preclinical studies, and studies that were not written in English.

Abstract and full-text text screening were conducted in duplicate by two independent reviewers (AG and NM) according to eligibility criteria outlined in the review protocol. All publications that explicitly mentioned the use of a tool to detect nociplastic pain or central sensitization in people with endometriosis-related pain were included. All discrepancies were resolved by a third party (EG) or discussion. Before screening, AG, NM, and EG reviewed five publications together to ensure a clear understanding of the eligibility criteria. The data collection form was jointly created by members of the team in order to determine variables that were meaningful for extraction. Data extraction was conducted by AG and NM, who used information on study demographics (i.e., country of origin, author, study type), assessment type (i.e., patient-reported, clinician-reported, etc.), the tool used for detecting nociplastic pain or central sensitization, and the study’s reasoning or psychometric properties if they were available.

We conducted a narrative synthesis of all included studies, which were grouped according to assessment type and the tool used. When we encountered a systematic review, we noted the type of assessment used to identify central sensitization or nociplastic pain in each study.

### 2.3. Results

**Figure 1:**
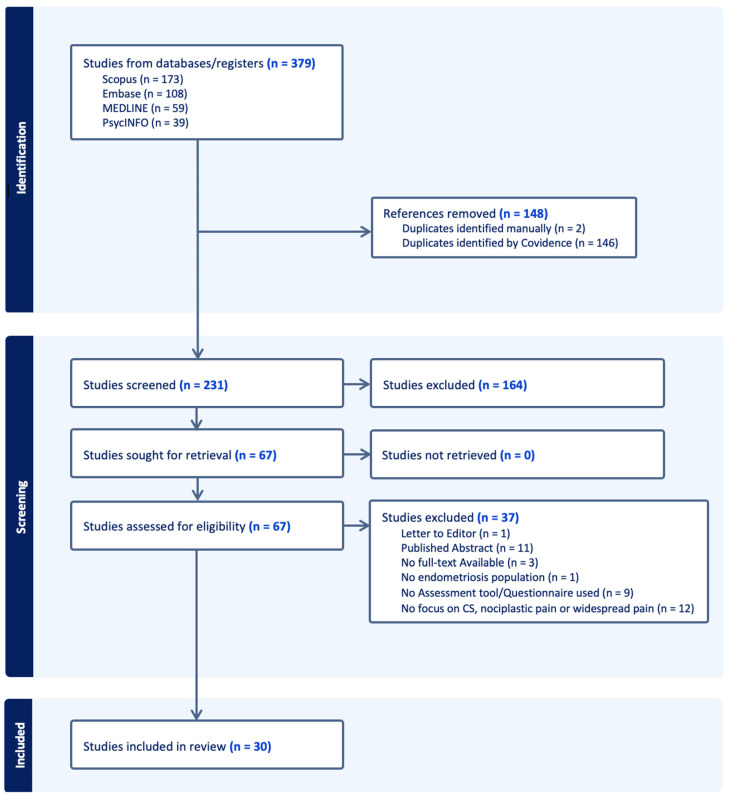
PRISMA flow diagram.

A total of 379 citations were retrieved from the databases. After removing 148 duplicates, 231 citations underwent title and abstract screening. Of these, 67 proceeded to full-text screening and were assessed for eligibility. All told, 37 studies were excluded for the following reasons: 1 was a letter to the editor, 11 were published abstracts, 3 did not have the full text available, 1 did not have an endometriosis population, 9 did not mention an assessment tool or questionnaire, and 12 did not focus on central sensitization (CS), nociplastic pain, or widespread pain. In total, 30 papers were included in the final review and underwent data extraction. The PRISMA flow diagram for study inclusion is shown in Figure 1. Of the 30 papers, 21 (70%) were primary research articles including cross-sectional (n = 12), cohort (n = 7), case–control (n = 1), and cross-over (n = 1) studies, where the method used to identify sample endometriosis participants with CS or nociplastic pain was clearly stated. The remaining 9 papers were reviews that explicitly stated a tool or assessment method used to identify CS, nociplastic pain, or widespread pain.

Most studies were from the United States (n = 8), Italy (n = 6), and Canada (n = 4). The remaining countries, each with fewer than 4 studies, were Australia, Brazil, China, Denmark, France, New Zealand, Spain, Sweden, and the United Kingdom. All studies referenced an adult population experiencing endometriosis-related chronic pelvic pain. A complete list of included papers and their general demographics is shown in Table 1. Approximately, half (14/30) of the studies reported the exclusive use of patient-reported questionnaires to identify CS and nociplastic pain. Conversely, 6 papers exclusively referenced the use of semi-objective assessments like QST to identify CS and nociplastic pain, with the pressure pain threshold (PPT) consistently used across all studies. Only 2 studies exclusively mentioned clinical assessments as a method of detecting CS: one focused on identifying abdominopelvic trigger points, while the other used the convergence pelvic and perineal (PP) criteria. Lastly, 27% (8/30) of the papers used mixed methods of identification. This involved the collective use of patient-reported questionnaires, including the PainDETECT questionnaire, the McGill questionnaire, the brief pain inventory, and the visual analogue scale (VAS), along with QST modalities and clinical assessments. A summary of the assessments identified in all 30 papers is shown in Table 2.

### 2.4. Discussion

#### 2.4.1. Patient-Reported Questionnaires

Given the complex pathophysiology of nociplastic pain and the mechanisms underlying CS development, detection through subjective assessments, such as questionnaires, is challenging. However, with increasing amounts of research identifying conditions with shared symptomatology, such as in chronic overlapping pain conditions (COPCs), questionnaire tools designed to differentiate based on CS-related symptoms are becoming more prevalent in clinical practice [61].

Most studies in this review exclusively used independent patient-reported questionnaires as proxies of centralized pain. The American College of Rheumatology (ACR) 2011 fibromyalgia survey score (FSS) was referenced as the sole indicator of CS [21,46,47,54], or was used alongside the CSI [56,59]. The fibromyalgia questionnaire assesses widespread pain using a 19-point body map and a 12-point symptom severity scale to give a total score out of 31. A score of ≥13/31 indicates fibromyalgia; however, recent studies suggest using the score as a continuous measure to assess the degree of CS present [46]. As-Sanie et al. (2021) [46] found a 27% increase in the odds of post-hysterectomy persistent pelvic pain with every 1-point increase in the pre-operative FSS. Endometriosis and fibromyalgia are both on the National Institutes of Health Pain Consortium list of COPCs. These conditions frequently co-occur and are postulated to share CS as their underlying mechanism of persistent pain [46,61]. Therefore, using the FSS as an indicator of CS—and by extension, the nociplastic pain phenotype—is promising in clinical settings involving both endometriosis and fibromyalgia. However, there are limited studies focusing exclusively on the use of the FSS in an endometriosis population.

The CSI was another questionnaire that was frequently used to identify CS in clinical settings [13,23,27,45,50,51,52,56,57,58,59,60]. This questionnaire has 25 items and focuses on the psychophysiological symptoms of daily living that indicate CS, such as sensitivity to light and restlessness, as well as more localized pain in areas such as the pelvis [62]. It scores patients from 0 to 100, with higher scores indicating a greater degree of CS [62]. Unlike the FSS, the CSI has a validated cut-off point (≥40/100) that indicates CS symptoms in an endometriosis pain population [27]. Orr et al. (2022, 2023) [23,27] demonstrated that endometriosis patients who scored above the 40-point cut-off on the CSI also have poorer patient-reported pain outcomes, indicating its potential use as a screening tool for pain outcomes. Similarly, using the CSI, Raimondo et al. (2023) [13] found that individuals with CS also had higher rates of first-line hormonal treatment failure compared to those without CS. Furthermore, in people with endometriosis, the CSI score was significantly associated with a number of COPCs, which in this paper were part of a wider category of termed central sensitization syndromes and pelvic pain-related comorbidities [23]. These syndromes/comorbidities have been shown to be independent predictors of quality of life following endometriosis surgery [22].

However, there has been speculation as to whether the CSI measures symptoms related to CS or simply the presence of a hypervigilant psychological state related to stress [59]. This theory is further supported by the strong association between CSI scores and psychological markers, like pain catastrophizing, depression, and anxiety [26]. Alternatively, the CSI has also shown significant correlations with QST measures like the pressure pain threshold (PPT) (r = −0.25 [95% CI: −0.28 to −0.21]) in chronic pain populations [63]. Therefore, there is a need for more research that explicitly looks at the relationship between the CSI and semi-objective assessments of somatosensory changes, like QST, in an endometriosis population.

Some studies noted the use of the PainDETECT questionnaire, a 7-item self-reported form asking patients to rate the quality, severity, and characteristics of their pain in order to assign a total score from −1 to 38 [64,65]. This questionnaire was initially developed in association with the German Research Network on Neuropathic Pain (DFNS) (Munich, Germany) to aid clinicians in detecting neuropathic-like pain in chronic lower back pain patients. It was found to have sensitivity and specificity values of 84%, with a cut-off point of ≥19/31 indicating a likely “neuropathic-like” component [64,66]. Since then, it has been used in other chronic-pain related studies including a cross-sectional study looking at women with endometriosis-associated pain, where the PainDETECT cut-off scores are used to group participants into those with nociceptive (≤12), mixed nociceptive and neuropathic pain (13–18), and neuropathic-like (≥19) pain; 40% were classified as neuropathic, 35% as mixed, and 25% as nociceptive [67]. They also showed that women classified as having “neuropathic-like” pain also reported more psychological distress (i.e., anxiety, depression, etc.) and higher pain intensity scores [67]. Moreover, there have been strong correlations between PainDETECT scores and FSSs (rho = 0.441, *p* < 0.001) [67]. Since fibromyalgia is considered a nociplastic pain condition, the strong association between the PainDETECT and FSS indicates that the PainDETECT questionnaire may have the ability to capture those with nociplastic pain. Within the same study, the Pain Sensitivity Questionnaire (PSQ) was also correlated with the FSS but was not found to have clinical significance despite previously showing positive correlations with experimental pain intensity rating in people with chronic pain [68] and endometriosis [69]. Whether the PSQ has clinical applications as a CS proxy in endometriosis populations is still to be determined.

Additionally, in a sample of women with endometriosis, bladder pain syndrome, and pelvic pain, Coxon et al. (2023) correlated the seven sensory-based questions from the PainDETECT questionnaire to QST modalities that measured the same stimulus–response relationship. The heat pain threshold (HPT) correlated significantly with the question “is cold or heat (bath water) in this area occasionally painful?” (r = 0.32, *p* = 0.032), the pressure pain threshold (PPT) correlated significantly with the question “does slight pressure in this area, e.g., with a finger, trigger pain?” (r =0.47, *p* < 0.001), and mechanical pain sensitivity (MPS) correlated significantly with the question “is light touching (clothing, a blanket) in this area painful?” (r = 0.38, *p* = 0.009) [4]. They also found that women in the endometriosis group described “painful attacks” as their most common sensory symptom [4]. 

Given its association with HPT, PPT, and MPS, as well as its moderate association with the FSS, the PainDETECT questionnaire may be a promising tool for classifying CS and the presence of nociplastic pain in endometriosis populations with mixed pain phenotypes. However, there are limited studies exclusively examining the neuropathic-like pain subgroup, which may include a subset with concurrent nociplastic pain and CS, using PainDETECT [67]. Therefore, additional investigation in an endometriosis pain population is needed to determine if the PainDETECT can distinguish those in the neuropathic pain group who exclusively have neuropathic pain from those with nociplastic pain features. 

Lastly, Phan et al. (2021) [48] used a cut-off >9/75 painful sites on the brief pain inventory (BPI) body map as an identifier of widespread pain. The BPI was created by the Pain Research Group of the World Health Organization (WHO) Collaborating Centre for Symptom Evaluation in Cancer Care to assess pain intensity and daily interference with life in cancer patients [70]. Phan et al. (2021) [48] only noted the use of the BPI body map in collaboration with the PPT and myofascial trigger points. Its short form has been used in endometriosis-associated pain assessments and has indicated minimal clinically important differences (MCIDs) after treatment [71]. However, like the visual analogue scale (VAS) and Numerical Scale Rating (NRS), which have also shown MCID in endometriosis pain populations [71], there are limited studies specifically looking at the BPI map or score, and any association with CS and nociplastic pain. 

It is important to note that the presence of CS in and of itself indicates altered CNS processing, and therefore, outside of their use as patient-reported outcome measures, simple NRS and VAS questionnaires asking patients to rate their general pain may not be reliable indicators of CS [9]. However, some studies have used a VAS in association with other questionnaires such as the McGill pain questionnaire to help identify widespread hyperalgesia [37].

#### 2.4.2. Semi-Objective Assessments

Quantitative Sensory Testing (QST) is an objective measure of altered somatosensation and CS. Standardized by the German Research Network on Neuropathic Pain (DFNS) in 2006, QST assesses sensory loss or gain and identifies patterns of large- and small-nerve fiber dysfunction through 13 different modalities [72]. Although the DFNS recommends using the full QST protocol, most studies in this review did not. Only Coxon et al. (2023) [4] used all 13 DFNS modalities, along with the PainDETECT questionnaire, in their “Translational Research in Pelvic Pain” study. The DFNS modalities include 1) thermal tests such as heat and cold detection and pain thresholds (HPT, HDT, CPT, CDT), thermal sensory limen (TSL), and paradoxical heat sensation (PHS), and 2) mechanical tests such as mechanical detection threshold (MDT), mechanical pain threshold (MPT), stimulus–response function including mechanical pain sensitivity (MPS) and dynamic mechanical allodynia (DMA), the wind-up ratio (WUR), vibrational detection threshold (VDT), and pressure pain threshold (PPT) [4,30].

Furthermore, when all 13 modalities are assessed together, the scores from each modality can be entered into a non-deterministic clustering algorithm and used to group patients into different sensory profiles: (1) sensory loss; (2) mechanism hyperalgesia; (3) thermal hyperalgesia; or (4) healthy [73]. A key feature of distinguishing CS from peripheral sensitization with QST is the use of a primary site, usually the site of local pain for detecting peripheral sensitization, and the use of a heterotopic site away from the primary noxious stimulus for CS [30]. Additionally, the assessment of sensory changes must be compared using reference values for the body site, age, and sex to ensure proper comparisons can be made [30,72]. The assessment of whether reported QST was properly standardized using reference data was outside the scope of this review; however, this may be a further research interest for studies investigating the validity of QST results in endometriosis populations.

However, other methods of pain stimulation and assessment were used in the papers reviewed, such as the electrical pain threshold test (EPT), which involved the application of electrical pulses using the PainMatcher (Cefar Medical AB, Lund, Sweden), and ischemic pain tests (IPTs), which measure the pain response following a reduction in blood flow to a limb [39,74]. Although there may be similarities between these tests and the 13 DFNS tests, it is important to note that they measure different mechanisms of pain. For instance, the PPT, which was the most commonly referenced semi-objective assessment, can detect CS in deep tissue as a result of pressure application, while the ischemic pain test measures the pain threshold as a result of reduced blood flow and lactic acid build-up in the muscles [4,39,72]. While there is sufficient evidence supporting the connection between PPT and CS in women with endometriosis, studies tying ischemic pain to CS in women with endometriosis are limited [4,69,75,76].

Despite the standardization of the QST methodology by the DFNS, many studies describe alternative ways to record and measure modalities. For example, some use a pressure cuff [40] instead of an algometer [37,38,43,48,49,69] for PPT determination. Another example is using the 1st, 5th, and 10th mechanical stimulus ratings to determine moderately painful stimuli for WUR as an alternative to the DFNS recommendation of 256 mN [40,72]. Therefore, it appears that even in an endometriosis pain sensitivity research setting, there is no standard method or equipment used to conduct QST or any of the associated modalities, as many deviate from the DFNS recommendations. This makes it difficult to compare results across studies and to reproduce findings.

#### 2.4.3. Clinician Assessments

Various physician-led clinical assessments were used to detect the presence of endometriosis-related sensitization. For instance, Aredo et al. (2017) [42] and Phan et al. (2021) [48] noted the use of a digital exam to identify active myofascial trigger points in the pelvic region as possible indicators of abdominopelvic tenderness and allodynia, which are proxies of sensitization. Somatosensory signaling from visceral and somatic organs sometimes converges on the same sensory neuron within the CNS [77] (pp. 175–177). This process is known as viscerosomatic convergence and provides an explanation for the pain experienced by some women with endometriosis [19,77]. However, this viscerosomatic neural interaction also explains how chronic visceral pain signals can sensitize afferent neurons in the spinal cord, resulting in symptoms of widespread hyperalgesia and allodynia [8,78]. Additionally, prolonged viscerosomatic convergence from pain signaling can lead to hypertrophy in skeletal muscles in the referred area, which can present itself as viscerosomatic reflexes like myofascial trigger points [79]. Aredo et al. (2017) [42] proposed examining the number and location of abdominopelvic myofascial trigger points, as well as the intensity rating of the pain elicited, as indicators of CS. Furthermore, some studies have linked the presence of active myofascial trigger points to sustained nociception and decreased pain thresholds following the removal of the initial noxious stimulus [8,79]. 

Following the examination, Aredo et al. (2017) [42] also conducted a semi-objective neuromusculoskeletal pain assessment of dermatome allodynia and hyperalgesia using a pinch-and-roll technique and a Wartenburg pinwheel, respectively. They also assessed myotome myofascial trigger points in regional and general muscles. However, although myofascial trigger points are frequently found in endometriosis patients and in those with other COPCs like irritable bowel syndrome, bladder pain syndrome, and vulvodynia, there is limited evidence supporting the belief that all endometriosis patients with these trigger points also have CS [12,80,81].

Alternatively, Cardaillac et al. (2023) used the convergence pelvic and perineal (PP) pain criteria, which were developed by an international panel of specialists to help facilitate CS detection in people with chronic pelvic pain [55,82]. The convergence PP criteria prompt clinicians to assess pelvic sensitivity and pain diffusion in the lower urinary and digestive tract, the genito-sexual tract, the mucocutaneous area, and the muscular system using a checklist of 10 items [82]. This clinical method has shown excellent specificity (87%) and sensitivity (95%), with a score ≥5/10 in people with pelvic perineal pain when compared to expert diagnosis, and has been used to identify sensitivity in various chronic pelvic pain populations [83].

## 3. Application to Endometriosis Clinical Care

In endometriosis, nociceptive pain arises from the endometriosis lesions themselves, which can be locally invasive, due in part to somatic driver mutations [84], and/or be associated with peripheral (local) neuroproliferation in the microenvironment. This is associated with the expression of inflammatory and neurotrophic factors by endometriosis-related and other cell types [84,85,86,87,88,89,90]. Although this suggests that there may be a correlation between these peripheral factors and pain symptoms, more sufficiently powered studies to investigate their influence on nociceptive pain are needed. As endometriosis is also an estrogen-dependent disease, nociceptive pain in endometriosis could be treated by hormonal suppression of the endometrial-like lesions themselves as well as the hypothalamic–pituitary–ovarian axis. In addition, the surgical excision of these endometrial-like lesions would be expected to improve nociceptive pain. Deeply invasive endometrial-like lesions that infiltrate peripheral nerves (e.g., sciatic) may also contribute to neuropathic pain in patients, which can be treated by hormonal suppression or surgical excision of the endometrial-like lesions themselves.

Unlike neuropathic and nociceptive pain, CS and nociplastic pain persist despite unclear evidence regarding tissue damage in the somatosensory system [9]. Consequently, other measures such as those reviewed in this article are needed to identify nociplastic pain in endometriosis patients. Furthermore, treating the central contributors of pain in endometriosis largely depends on identifying the mechanism of sensitization development: is it ‘bottom-up,’ where peripheral influences and nociception drive CNS neuroplasticity, ultimately resulting in nociplastic pain, or is it ‘top-down,’ where pain amplification in the CNS exists independently of peripheral influences [91]? Nonetheless, endometriosis patients with a significant nociplastic pain component may not respond to hormonal or surgical treatments alone, and an interdisciplinary approach that addresses multiple pain contributors, whether local, central or psychosocial, may be more appropriate for pain management [92,93]. In a randomized controlled trial comparing an interdisciplinary pain treatment plan to standard peripheral pain treatments in women with chronic pelvic pain, 75% of patients in the multidisciplinary pain program reported improvements in their overall pain after one year, compared to 41% in the standard treatment group (*p* < 0.01) [94]. Additionally, interdisciplinary care models have historically shown significant effects in terms of improving patient-reported pain outcomes in people with chronic lower back pain, a known COPC, compared to standard monotherapies [95,96,97,98].

Many of these interdisciplinary approaches encompass pain neuroscience education, which includes pain beliefs and pain catastrophizing, validates pain despite the absence of positive test results, and explains the influences of psychological and social aspects on pain perception. It has been attributed to reduced pain and disability, and improved function and movement [99,100]. Health behavior changes, such as the adoption of diets that target comorbidities, such as irritable bowel syndrome and painful bladder syndrome [17,101,102], and the consumption of whole foods [103,104] have also been found to be effective in reducing pain. Psychological treatments such as cognitive behavioral therapy (CBT), mindfulness, meditation, and acceptance and commitment therapy can lead to improved mood, function, self-efficacy, and reduced pain and catastrophizing in people with rheumatoid arthritis [105]. CBT can also be effective in treating the adverse effects of chronic pain, such as insomnia and sexual dysfunction [106]. Additionally, site specific manual physiotherapy has been proven to significantly improve dysmenorrhea and dyspareunia in women with endometriosis [107]. It has also shown a significant effect on global response assessment, pain intensity, quality of life, fear avoidance, and disability in women with CPP [108,109,110,111]. There may also be a role for myofascial trigger point injections, treatments targeted to functional bladder and bowel pain, and medications that modulate the nervous system [112].

## 4. Limitations

This scoping review was limited by the novelty of the nociplastic pain phenotype within the research and clinical communities. The IASP only recently published definitions and guidelines for ‘possible’ and ‘probable’ nociplastic pain clinical identification [2]. Clinical terminology is still evolving as clinicians and researchers work to better identify nociplastic pain and CS [6,9]. As a result, we may have missed some studies related to endometriosis pain that investigated nociplastic pain using different terms. Additionally, although recent evidence supports the use of biomarker identification in endometriosis pathology [113], we did not capture biomarker measures of nociplastic pain in this review. Another limitation was the inclusion criteria, which allowed the use of all study types, including review articles. The inclusion of review articles may have hindered comparisons across the different articles. Furthermore, we utilized a narrative analysis to identify tools and methods of assessments. While this allows for a comprehensive qualitative synthesis and interpretation of the available literature, it also inherently introduces a degree of selection and interpretation bias, modulated by reviewer expertise. Additionally, many studies were of mixed pelvic pain groups, that included endometriosis patients, which prevented further analysis of population characteristics across studies.

## 5. Conclusions

This review provided a glimpse into the breadth of assessments used to identify evidence of CS and the presence of the nociplastic pain phenotype in individuals with endometriosis-related pain. Multiple tools were used, including patient-reported questionnaires such as the CSI, FSS, and PainDETECT, as well as clinician assessments of trigger points in the abdominopelvic area and QST. However, the ability of these tools to accurately identify CS and nociplastic pain within an endometriosis population is still under investigation. More primary research is needed to elucidate the mechanisms of CS in endometriosis and provide a core set of validated clinically and feasible methods of identifying both CS and nociplastic pain in endometriosis.

## Figures and Tables

**Table 1:** Study characteristics.

Authors, Year.		Country	Study Type	Assessment Type	Methods for CS/Nociplastic Detection
1	Bajaj et al., 2003 [37]	Denmark	Case–control	Mixed	VAS and McGill pain questionnaire + body map Pressure pain threshold (PPT) Tactile threshold
2	Laursen et al., 2005 [38]	Denmark	Cross-sectional	Semi-objective	Pressure pain threshold (PPT)
3	He et al., 2010 [39]	China	Prospective cohort	Mixed	Electrical pain threshold (EPT) Ischemic pain tests (IPTs) Short-form McGill pain questionnaire
4	Napadow et al., 2012 [40]	United States	Cross-over	Semi-objective	Pressure pain threshold (PPT) Wind-up ratio (WUR) Diffuse noxious inhibitory control (DNIC)
5	Giamberardino et al., 2014 [41]	Italy	Narrative review	Semi-objective	Somatic pain thresholds Electrical pain threshold (EPT) Ischemic pain tests (IPTs)
6	Aredo et al. 2017 [42]	United States	Narrative review	Clinical examination	Digital exam of myofascial trigger points in abdominopelvic area coupled with neuromusculoskeletal exam
7	Costantini et al., 2017 [43]	Italy	Prospective cohort	Semi-objective	Pressure pain threshold (PPT) Electrical pain thresholds (EPT)
8	Grundstrom et al., 2019 [44]	Sweden	Cross-sectional	Semi-objective	Heat pain threshold (HPT) Cold pain threshold (CPT) Pressure pain threshold (PPT)
9	Orr et al., 2020 [45]	Canada	Cross-sectional	Mixed	Central sensitization inventory (CSI) + Digital exam for bladder/pelvic floor tenderness
10	As-Sanie et al., 2021 [46]	United States	Prospective cohort	Patient-reported questionnaire	Fibromyalgia survey score (FSS)
11	Evans et al., 2021 [47]	Australia	Cross-sectional	Patient-reported questionnaire	Fibromyalgia survey score (FSS)
12	Phan et al., 2021 [48]	United States	Cross-sectional	Mixed	Brief pain inventory (BPI) Pressure pain threshold (PPT) Digital exam of myofascial trigger points in abdominopelvic area coupled with neuromusculoskeletal exam
13	Shafrir et al., 2021 [49]	United States	Cross-sectional	Semi-objective	Pressure pain threshold (PPT)
14	De Arruda et al., 2022 [50]	Brazil	Cross-sectional	Patient-reported questionnaire	Central sensitization inventory (CSI)
15	Green et al., 2022 [51]	United States	Narrative review	Patient-reported questionnaire	Central sensitization inventory (CSI)
16	Orr et al., 2022 [27]	Canada	Prospective cohort	Mixed	Central sensitization inventory (CSI) Central sensitivity syndromes and other pelvic pain related comorbidities (CSSs)
17	Ryan et al., 2022 [52]	New Zealand	Cross-sectional	Patient-reported questionnaire	Central sensitization inventory (CSI)
18	Simpson et al., 2022 [53]	Australia	Systematic review	Mixed	Ischemic pain test (IPT) Electrical pain test (EPT) McGill pain questionnaire + VAS Wind-up ratio (WUR) Mechanical pain threshold (MPT) Digital exam of myofascial trigger points in abdominopelvic area Central sensitivity syndromes and other pelvic pain related comorbidities (CSSs)
19	Till et al., 2022 [54]	United States	Narrative review	Patient-reported questionnaire	Fibromyalgia survey score (FSS)
20	Cardaillac et al., 2023 [55]	France	Prospective cohort	Clinical examination	Convergence PP criteria (10 clinical criteria)
21	Cetera et al., 2023 [56]	Italy	Narrative review	Patient-reported questionnaire	Central sensitization inventory (CSI) Fibromyalgia survey score (FSS)
22	Cetera et al., 2023 [57]	Italy	Literature review	Patient-reported questionnaire	Central sensitization inventory (CSI)
23	Coxon et al., 2023 [18]	United Kingdom	Narrative review	Mixed	DFNS 13 QST Modalities PainDETECT Questionnaire
24	Orr et al., 2023 [23]	Canada	Prospective cohort	Patient-reported questionnaire	Central sensitization inventory (CSI)
25	Quintas-Marques et al., 2023 [58]	Spain	Cross-sectional	Patient-reported questionnaire	Central sensitization inventory (CSI)
26	Raimondo et al., 2023 [13]	Italy	Cross-sectional	Patient-reported questionnaire	Central sensitization inventory (CSI)
27	Till et al., 2023 [21]	United States	Cross-sectional	Patient-reported questionnaire	Fibromyalgia survey score (FSS)
28	Cetera et al., 2023 [59]	Italy	Systematic review	Patient-reported questionnaire	Central sensitization inventory (CSI) Fibromyalgia survey score (FSS)
29	Coxon et al., 2023 [4]	United Kingdom	Cross-sectional	Mixed	DFNS 13 QST Modalities PainDETECT Questionnaire
30	Liu et al., 2024 [60]	Canada	Prospective cohort	Patient-reported questionnaire	Central sensitization inventory (CSI)

**Table 2:** Summary of assessments/tools for nociplastic pain in endometriosis.

Clinical
Patient-Reported Outcomes and Physical Examination
Central sensitization inventory (CSI); Fibromyalgia survey score (FSS); PainDETECT; Brief pain inventory (BPI); McGill pain questionnaire; Visual analog scale (VAS); Central sensitivity syndromes (CSS) or Chronic overlapping pain conditions (COPC); Digital exam of myofascial trigger points (e.g., abdominal); Digital exam for (bladder +) pelvic floor tenderness; Convergence PP criteria.
**Research**
**Sensory Testing** Quantitative sensory resting (QST) ^1^; Diffuse noxious inhibitory control (DNIC); Electrical pain threshold (EPT); Ischemic pain tests (IPTs).

^1^ DFNS QST protocol [30].

## Data Availability

Data sharing is not applicable to this article.

## Supplementary Materials

The following supporting information can be downloaded at: https://www.mdpi.com/article/10.3390/jcm13247521/s1, Table S1: Ovid MEDLINE (R) ALL <1946 to 22 April 2024>; Table S2: Preferred Reporting Items for Systematic reviews and Meta-Analyses extension for Scoping Reviews (PRISMA-ScR) Checklist.
